# Feeding flaxseed to chicken hens changes the size and fatty acid composition of their chicks’ brains

**DOI:** 10.3389/fphys.2024.1400611

**Published:** 2024-06-07

**Authors:** Rosemary H. Whittle, Elijah G. Kiarie, David W. L. Ma, Tina M. Widowski

**Affiliations:** ^1^ Department of Animal Biosciences, University of Guelph, Guelph, ON, Canada; ^2^ Campbell Centre for the Study of Animal Welfare, University of Guelph, Guelph, ON, Canada; ^3^ Department of Human Health and Nutritional Sciences, University of Guelph, Guelph, ON, Canada

**Keywords:** chicken, broiler, layer, maternal diet, omega-3 fatty acids, brain size

## Abstract

Diets fed to commercial chicken breeders are high in n-6 fatty acids (n-6 FAs) and low in n-3 fatty acids (n-3 FAs). N-3 FAs are essential for embryonic brain development. In precocial birds, like chickens, brain development and brain n-3 FA accrual occur primarily before hatching. In two experiments, broiler and layer breeders were fed diets with or without flaxseed as the source of n-3 FAs from plant-based alpha-linolenic acid. Day-old broiler (*n* = 80) and layer (*n* = 96) offspring were dissected to calculate the percentage brain-to-body weight. Brain FA analyses from total lipid extracts were determined in the broiler (*n* = 24) and layer (*n* = 24) offspring brains, and the percentage FA composition and concentration (µg FAs per g brain) were calculated for each n-3 and n-6 FA. The brain size was only increased in broiler offspring from mothers fed flaxseed (χ2 = 9.22, *p* = 0.002). In layer offspring only, the maternal flaxseed diet increased the brain concentration and percentage of n-3 FAs and decreased n-6 FAs (*p* < 0.05). We showed that feeding flaxseed to mothers increased the brain size in broiler offspring and altered brain FA composition in layer offspring. These results may have implications for poultry and other captive bird species fed diets low in n-3 FAs.

## 1 Introduction

Long-chain omega-3 polyunsaturated fatty acids (n-3 FAs) are key structural components of neural, muscle, and retinal tissue in vertebrate species, including fish (Závorka et al., 2022), mammals (Carlson and Neuringer, 1999), and birds (Speake et al., 1998). The two predominant n-3 FAs are docosahexaenoic acid (DHA) from marine dietary sources such as fish oil and its precursor alpha-linolenic acid (ALA) from plant sources, including flaxseed (Innis, 2008; Bradbury, 2011). While n-3 FAs are important, omega-6 FA (n-6 FA) cannot be overlooked. n-3 FA and n-6 FA compete for the same metabolic enzymes. When n-6 FA and n-3 FA consumption is imbalanced (high n-6 FA-to-n-3 FA ratio), more n-6 FAs than n-3 FAs are found in cellular membranes (Schmitz and Ecker, 2008). Dietary n-3 FAs, particularly DHA, are essential in maternal and postnatal diets for embryonic brain and central nervous system development. Perinatal n-3 FA nutrition affects both brain size and fatty acid composition of the tissues (Coti Bertrand et al., 2006). For example, higher consumption of prey rich in n-3 FAs in wild brown trout resulted in increased muscle DHA concentration, positively correlated with relative brain size (Závorka et al., 2022). A study on neurogenesis in embryonic rats found that n-3 FA deficiency during development resulted in reduced brain size due to reductions in the size of the cortical plate, primordial hippocampus, and dentate gyrus. In addition to neurogenesis, other processes in the brain rely on DHA, including neurotransmission and protection against oxidative stress, with n-3 FA deprivation affecting the membrane stability and gene expression (Innis, 2007). In rats, dietary n-3 FAs are involved in the upregulation and downregulation of genes affecting synaptic plasticity, signal transduction, energy metabolism, and neurotransmitter receptors (Kitajka et al., 2004; Kuperstein et al., 2005). In one study, for example, n-3 FA-deficient rat dams produced offspring with impaired dopaminergic regulatory systems (Kuperstein et al., 2008). Furthermore, perinatal n-3 FA has been linked to cognitive ability in many species, including mammals (Fedorova and Salem, 2006; Chung et al., 2008) and birds (Lamarre et al., 2021). For example, mice (Carrié et al., 2000) and rats (Chung et al., 2008) from mothers deficient in n-3 FAs had longer escape latencies in the Morris water maze test. Furthermore, red-legged partridges that were n-3 FA-deficient produced offspring with decreased discrimination ability (Fronte et al., 2008).

In birds, the maternal n-3 FA status influences offspring development through the egg nutrient content and, most importantly, the egg yolk (Cherian, 2011). The nutrient content of bird eggs varies, with altricial birds laying eggs with small dilute yolks compared to precocial birds, whose eggs have large yolks consisting of a high percentage of lipids and proteins (as reviewed in (Speake and Wood, 2005)). In precocial birds, such as chickens, the developmental period within the egg is when DHA accumulation in the brain and other tissues is the most significant (Speake, 2005). Facilitating brain development through dietary n-3 FAs may positively influence cognitive ability. Commercial chickens are fed cost-effective diets primarily composed of grains, including corn and soybean. However, these diets have a high n-6-to-n-3 FA ratio, resulting in eggs with n-6:n-3 FA ratios averaging between 9:1 (EU) and 19.5:1 (USA) (De Meester, 2008). These ratios exclude eggs from commercial hens fed n-3 FA-rich diets for omega-3 egg niche markets. The current recommendations for chicken breeder diets are based on optimizing production outcomes (i.e., poultry meat and egg) with least-cost diet formulations, where brain health and development are not considered. However, adequate levels of n-3 FAs in the egg may be necessary. The ideal ratio of n-6 to n-3 FAs in chicken eggs to promote embryonic brain development is unknown. Research shows that free-ranging chickens eating a wide variety of vegetation lay eggs with a ratio close to 1:1 (Simopoulos and Salem, 1989). Considering the importance of n-3 FAs in embryonic development, feeding potentially n-3 FA-deficient commercial diets to breeder chickens may have long-term negative implications for the behavior and welfare of their offspring.

To the best of our knowledge, only one study has examined the effect of maternal-fed n-3 FAs (from marine oils) on chicken brain size at hatching and did not show increased brain size in broiler chicken offspring from supplemented mothers (Ajuyah et al., 2003). Broiler chickens, raised for meat, and egg-laying chickens are phenotypically very different due to their selection for different production traits. Broiler chickens have been genetically selected for large appetites, fast growth, muscle gain, and feed efficiency to rapidly produce meat for human consumption (Bradshaw et al., 2002). On the other hand, egg-laying chickens have been selected for reproductive efficiency, producing many eggs with characteristics desirable for human consumption (Anderson et al., 2013). Selection for these traits has resulted in broiler and egg-laying chickens differing physically, behaviorally, and metabolically. For example, mothers of broiler chickens lay eggs with larger yolks and higher energy availability than layer breeders (Nangsuay et al., 2015). Broiler-chick embryos also grow faster and have larger organ weights at hatching than egg-layer chicks, likely due to their selection for growth and increased nutrient availability in the eggs (Nangsuay et al., 2015). In egg-laying chickens, there are differences between genetic strains, primarily brown *versus* white egg-laying phenotypes, which also typically correspond with feather color. Strain-dependent effects have been found for n-3 FA deposition in hatching eggs. Shaver White hens deposited a higher percentage of DHA into the phospholipid fraction of the egg yolk than ISA Brown hens, 40.8% *versus* 19%, respectively (Akbari Moghaddam Kakhki et al., 2020b). Akbari Moghaddam Kakhki et al. (2020b) also found that white-strain chick embryos utilized 11% more phospholipids during embryonic development, with chicks from breeders fed diets enriched with ALA utilizing less ALA and more DHA than chicks from control-fed breeders. These findings suggest that phylogeny affects FA metabolism in chickens.

This study aimed to investigate the effects of maternal diets supplemented with plant-based n-3 FAs on offspring brain size and brain FA composition using two different avian models. To accomplish this, we conducted two experiments. We fed one strain of broiler breeder (experiment 1) and two strains of layer breeders (experiment 2), one brown and one white egg-producing, diets supplemented with n-3 FAs and compared brain measures in their offspring with those of chicks from mothers fed standard commercial poultry diets. We hypothesized that mothers supplemented with n-3 FAs would produce offspring with altered brain FA composition and brain size.

## 2 Materials and methods

All animal use and procedures in this study were considered and approved by the University of Guelph Animal Care Committee and followed the Canadian Council on Animal Care guidelines (Animal Utilization Protocol #4246).

### 2.1 Breeders and diet

Two successive experiments were performed to assess the effect of maternal flaxseed diets on the brain size and n-3 FA composition of broiler (experiment 1) and layer (experiment 2) offspring.

In experiment 1, eight pens of Ross 708 broiler breeder hens (*n* = 213) were fed a control (commercial diet formulated for broiler breeders) *versus* n-3 FA-supplemented diet during rearing and a control or n-3 FA-supplemented diet during laying, resulting in a 2 × 2 factorial design. The n-3 FA-supplemented diets contained 2.57% (wt/wt%) of an ALA-rich co-extruded full-fat flaxseed product (LinPRO, O&T Farms, Regina, SK, Canada). The n-6:n-3 FA ratio of the control diet during the laying period was 27.6:1 compared to the flaxseed diet ratio of 4.2:1. These dietary treatments resulted in four rearing–laying maternal diet combinations (MDCs): control–control, control–flaxseed, flaxseed–control, and flaxseed–flaxseed. Eggs were collected for incubation from each MDC at 30 and 33 weeks of age (WoA).

In experiment 2, two strains of layer breeders, Shaver White (*n* = 192) and ISA Brown (*n* = 192), were raised in 16 pens (4 per strain/treatment) and fed either a control (commercial diet formulated for laying hens) or a flaxseed-supplemented diet using the same product as in the broiler breeder diets. The n-6:n3 FA ratio of the control diet during the laying period was 14.7:1 compared with 5.3:1 for the flaxseed diet. These treatments resulted in four strain and maternal diet combinations: brown–control, brown–flaxseed, white–control, and white-flaxseed. At 30 and 36 WoA, eggs from each strain and maternal diet combination were collected for incubation.

**FIGURE 1:**
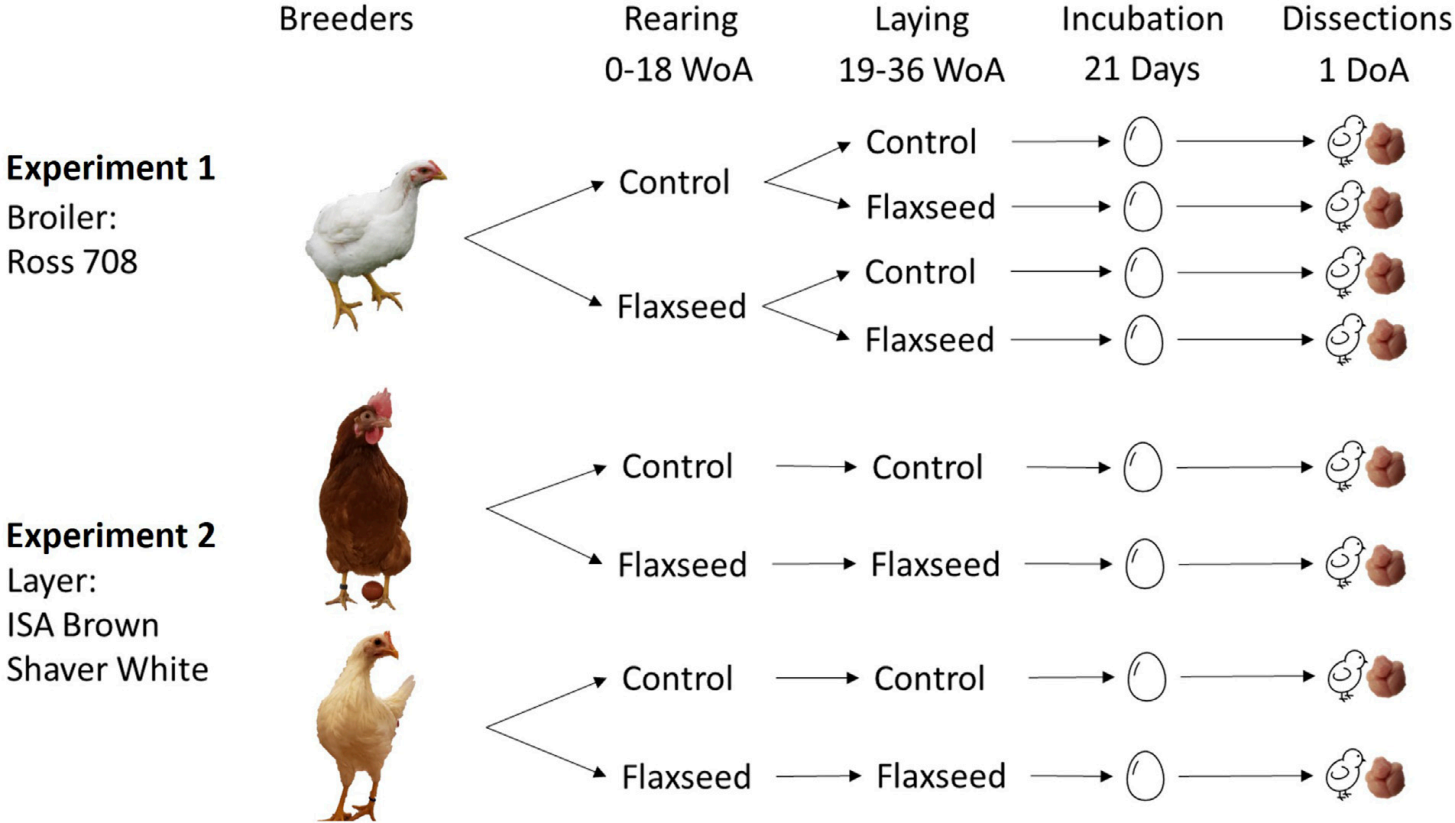
Experimental design for experiments 1 and 2. Experiment 1 used one strain of broiler breeder (Ross 708) fed control or flaxseed-supplemented diets in the rearing (0–18 WoA) and/or laying period (19–36 WoA), resulting in four rearing–laying diet combinations. Experiment 2 used two strains of layer breeders, one that produces brown-feathered offspring (ISA Brown) and one that produces white-feathered offspring (Shaver White), that were fed either control or flaxseed-supplemented diets during both the rearing and laying periods. Eggs were collected from the breeders at two different ages and incubated for 21 days. Upon hatching, unsexed chicks were euthanized for brain dissections.

Figure 1 shows the experimental designs and the different diet combinations for broilers and layer breeders. All diets were isocaloric, isonitrogenous, and specifically formulated to only differ in fatty acid composition. Detailed ingredients and calculated nutrients for broiler and layer breeders, as well as breeder body weight, egg weight, and chick weight, are found in the study by Whittle et al. (2024). There was no reported effect of the maternal flaxseed diet on breeder body weight, egg weight, or chick hatching weight.

### 2.2 Egg yolk fatty acid analysis

In experiment 1, three eggs were collected from each broiler breeder parent pen at 28 WoA. The egg yolks were combined and homogenized to take a pooled sample from each pen. Thus, one homogenized sample from each parent pen was assayed. Samples were sent to Activation Laboratories Ltd. (Ancaster, ON, Canada) for fatty acid–total lipid analysis, according to O’Fallon et al. (2007). Yolk fatty acids are reported as the mean percentage composition and standard error of the mean. The yolk n-6-to-n-3 FA ratio was calculated as Σ n-6 FA divided by Σ n-3 FA.

Extensive fatty acid analyses of eggs from broiler breeders and layer breeders of the same strains and fed the same product and identical diet formulations as in the current study have been previously reported (Akbari Moghaddam Kakhki and Kiarie, 2021; Thanabalan, 2023).

### 2.3 Offspring brain collection

Live hatch weight was taken for 40 male and 40 female broiler chicks from each MDC (N = 80) in experiment 1 and 108 female layer chicks from each strain by maternal diet combination (N = 108) in experiment 2. The sample size was calculated to determine the number of individuals needed for dissections by comparing the sum of the degrees of freedom to the 3R reduction (minimum number) and refinement (optimal number) principles. The minimum number of individuals was used (reduction) whilst still allowing analyses of population variation (Eisenhauer, 2008; Pandey and Bright, 2008). Ten unsexed broiler chicks and six unsexed layer chicks from each parent pen and parental age were euthanized using CO_2_ for dissections (broiler N = 80 and layer N = 96). The whole body and brain weights were recorded. Dissected brains were placed in labeled sample bags and flash-frozen over dry ice within 10 min of euthanasia. The samples were transferred to a −20℃ freezer until analysis. Brain size was calculated as the brain-to-body weight percentage (brain weight divided by body weight) multiplied by 100, for both experiments.

### 2.4 Offspring brain fatty acid analysis

For experiment 1, six chick brains were collected from each broiler breeder maternal diet combination balanced over breeder age (i.e., 30 and 33 WoA) and parental pen (N = 24). Similarly, for experiment 2, six brains were collected from each layer breeder strain and maternal diet combination balanced over breeder age (30 and 36 WoA) and parental pen (N = 24). For both experiments, brain fatty acids were analyzed by gas–liquid chromatography (Akbari Moghaddam Kakhki et al., 2020a). Each brain was homogenized in 1 mL of 0.1 M of KCl, and 100 µL of the homogenized brain was further diluted in 900 µL 0.1 M of KCl. A known concentration of C17:0 (50 µg) was added as an internal standard. Then, 4 mL of CHCl_3_:MeOH (2:1) was added and vortexed, and tubes were flushed with N_2_ and refrigerated at 4°C overnight. Samples were centrifuged at 357 *x g* (RCF) for 10 min, the chloroform layer was drawn off and dried down under a stream of N_2_, and then, 2 mL of 0.5 M KOH in MeOH was added before being saponified at 100°C for 1 h. To the cooled samples, 2 mL of hexane and 2 mL of 14% BF3-MeOH were added, and the samples were then methylated at 100°C for 1 h. Two mL of double-distilled water was added to the cooled samples to stop methylation. The samples were vortexed and centrifuged at 357 *x g* (RCF) for 10 min. The hexane layer was drawn off, dried under N_2_, and reconstituted in 500 µL of hexane for gas chromatography analysis and compared to the known standard (C17:0 50 µg) (Akbari Moghaddam Kakhki et al., 2020a). The percentage of fatty acids in the brain was calculated by (FA area/total area) * 100. Brain FA concentrations were calculated by determining the µg of FAs in 100 µL of the sample (FA area * standard (50 µg))/area standard. Values were then used to calculate (µg of FAs in 100 µL of sample/brain weight in 1 mL of solution) µg of FAs per g brain tissue accounting for different amounts of brain tissue added at the beginning of the process. The n-6-to-n-3 FA ratio was calculated by summing the area under the curve of all n-6 FAs and then all n-3 FAs. The ratio was determined by dividing the sum µg of n-6 FAs by the sum µg of n-3 FAs.

### 2.5 Statistics

Statistical analyses were conducted in R v4.1.2 and R Studio using the “stats,” “lme4,” and “emmeans” packages. Broiler breeder yolk FA content was analyzed using two-way ANOVA with maternal rearing and maternal laying diet as fixed effects. Significant interactions between fixed effects were explored using *post hoc* Tukey’s test. Brain-to-body weight ratio data were analyzed using linear mixed-effects models using the individual chick as the experimental unit. Maternal rearing and maternal laying diet were the fixed effects in experiment 1 for broiler offspring, and maternal diet and strain were the fixed effects in experiment 2 for layer offspring. Individual brain n-3 and n-6 fatty acids, Σ n-3 and Σ n-6 fatty acids, and n-6-to-n-3 FA ratio were analyzed with two-way ANOVA using maternal rearing and laying diet as fixed effects in experiment 1 for broiler offspring, and maternal diet and strain as fixed effects in experiment 2 for layer offspring and using the individual chick as the experimental unit. Significant interactions between main effects were analyzed using *post hoc* Tukey’s tests using Bonferroni correction for multiple testing.

## 3 Results

### 3.1 Broiler breeder yolk fatty acids

There was an interaction between maternal rearing and maternal laying diet for ALA (F = 5.69, *p* < 0.001), DHA (F = 5.31, *p* = 0.05), and Σ n-3 (F = 6.62, *p* = 0.03) (Table 1). Eggs from hens fed flaxseed in both the rearing and laying periods deposited the highest ALA, DHA, and total n-3 FAs; control–control hens deposited the least. Significant pairwise comparisons between rearing and laying maternal diet are given in Table 1. The maternal laying diet significantly affected the percentage of Σ n-6 (F = 6.77, *p* = 0.03). Eggs from hens fed the control diet (29.36% ± 0.52%) during the laying period had a higher percentage of n-6 FAs than those fed flaxseed (27.68% ± 0.32%). There was a significant effect of rearing (F = 9.13, *p* = 0.02) and laying diet (F = 79.81, *p* < 0.001) on the n-6-to-n-3 FA ratio in egg yolks. The ratio of n-6 to n-3 FAs was lower in hens fed flaxseed in the rearing (7.89 ± 1.24) and laying period (5.88 ± 0.41) compared with those fed control diets in the rearing (9.96 ± 1.37) and laying period (11.97 ± 0.71). A complete list of results for broiler breeder yolk fatty acids is given in Table 1.

**TABLE 1:** Yolk fatty acid analyses for eggs from broiler breeder hens fed either a control- or flaxseed-enriched diet during the rearing or laying period. Data are shown as % of total fatty acids. Significant pairwise comparisons (*p* < 0.05) between eggs laid by hens fed different rearing and laying diets are indicated with differing superscript letters.

Rearing diet Fatty acid↓/laying diet	Control Control	Flaxseed Control	Control Flaxseed	Flaxseed Flaxseed	Rearing	ANOVA Laying	Rearing*laying
Alpha-linolenic acid (C18:3 n-3)	1.14 ± 0.12^a^	1.32 ± 0.13^a^	2.20 ± 0.15^b^	3.07 ± 0.05^c^	***F = 13.18, p = 0.007***	***F = 94.40, p<0.001***	***F = 5.69, p<0.001***
Eicosatrienoic acid (C20:3 n-3)	0.08 ± 0.05	0.02 ± 0.00	0.08 ± 0.04	0.06 ± 0.01	*F = 1.16, p = 0.31*	*F = 0.31, p = 0.59*	*F = 0.31, p = 0.59*
Eicosapentaenoic acid (C20:5 n-3)	0.01 ± 0.004	0.03 ± 0.007	0.05 ± 0.003	0.08 ± 0.016	***F = 9.33, p = 0.02***	***F = 32.19, p<0.001***	*F = 0.19, p = 0.67*
Docosapentaenoic acid (C22:5 n-3)	0.18 ± 0.005	0.22 ± 0.019	0.32 ± 0.025	0.45 ± 0.077	*F = 2.74, p = 0.14*	***F = 13.00, p = 0.007***	*F = 0.77, p = 0.41*
Docosahexaenoic acid (C22:6 n-3)	0.87 ± 0.07^a^	1.10 ± 0.02^a^	1.46 ± 0.10^b^	1.89 ± 0.03^c^	***F = 44.96, p<0.001***	***F = 203.01, p<0.001***	***F = 5.31, p = 0.05***
Σ n-3	2.31 ± 0.15^a^	2.69 ± 0.30^a^	4.12 ± 0.24^b^	5.55 ± 0.04^c^	***F = 19.97, p = 0.002***	***F = 131.00, p<0.001***	***F = 6.62, p = 0.03***
Linoleic acid (C18:2 n-6)	25.70 ± 0.74	24.90 ± 0.39	23.66 ± 0.20	24.57 ± 0.70	*F = 0.01, p = 0.93*	*F = 3.08, p = 0.12*	*F = 1.59, p = 0.24*
Gamma-linolenic acid (C18:3 n-6)	0.55 ± 0.15	0.43 ± 0.07	0.40 ± 0.09	0.39 ± 0.36	*F = 0.28, p = 0.61*	*F = 0.59, p = 0.46*	*F = 0.18, p = 0.24*
Eicosadienoic acid (20:2 n-6)	0.55 ± 0.17	0.40 ± 0.06	0.43 ± 0.14	0.36 ± 0.06	*F = 0.69, p = 0.43*	*F = 0.39, p = 0.55*	*F = 0.09, p = 0.77*
Eicosatrienoic acid (20:3 n-6)	0.41 ± 0.19	0.22 ± 0.02	0.36 ± 0.13	0.22 ± 0.04	*F = 1.35, p = 0.28*	*F = 0.03, p = 0.86*	*F = 0.04, p = 0.84*
Arachidonic acid (20:4 n-6)	2.87 ± 0.09	2.68 ± 0.09	2.62 ± 0.14	2.31 ± 0.02	*F = 6.48, p = 0.06*	***F = 0.83, p = 0.03***	*F = 0.26, p = 0.63*
Docosadienoic acid (22:2 n-6)	0 ± 0	0.003 ± 0.003	0.04 ± 0.03	0 ± 0	*F = 0.83, p = 0.39*	*F = 0.83, p = 0.39*	*F = 1.17, p = 0.31*
Σ n-6	30.08 ± 0.81	28.64 ± 0.27	27.51 ± 0.25	27.86 ± 0.57	*F = 0.72, p = 0.42*	***F = 6.77, p = 0.03***	*F = 1.94, p = 0.20*
Σ n-6:Σ n-3	13.16 ± 0.67	10.78 ± 0.79	6.75 ± 0.40	5.02 ± 0.08	***F = 9.12, p = 0.02***	***F = 79.81, p<0.001***	*F = 0.22, p = 0.65*

Bold values represent significant values at *p* ≤ 0.05.

### 3.2 Brain size

**FIGURE 2:**
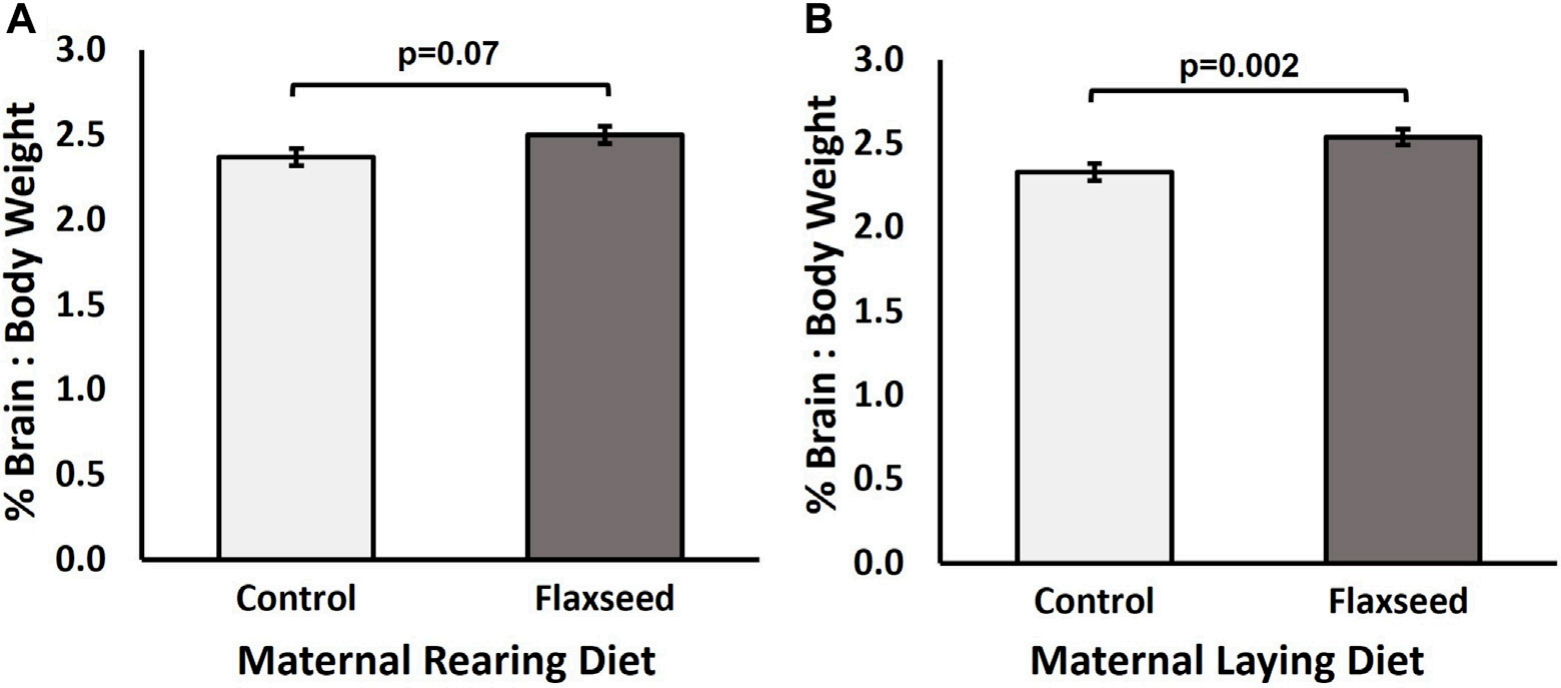
Experiment 1. Estimated mean (±SE) brain-to-body weight percentage [(brain/body weight) * 100] of day-old Ross 708 broiler chicks from breeders fed either a control or flaxseed diet during the rearing or laying period. **(A)** Maternal rearing diet tended to affect the brain-to-body weight percentage of the offspring at hatching (χ^2^ = 3.29, *p* = 0.07). **(B)** Maternal laying diet significantly affected the brain-to-body weight percentage of the offspring at hatching (χ^2^ = 9.22, *p* = 0.002).

Experiment 1: Broiler chicks from the flaxseed maternal rearing diet tended to have a larger brain-to-body weight percentage than chicks from the control maternal rearing diet treatment (χ^2^ = 3.29, *p* = 0.07; Figure 2A). The maternal laying diet significantly affected the brain-to-body weight percentage (χ^2^ = 9.22, *p* = 0.002) of the broiler offspring, with chicks from the flaxseed maternal laying diet having a larger brain-to-body weight percentage than chicks from the control maternal laying diet (Figure 2B). There was no interaction between maternal rearing and maternal laying diets (χ^2^ = 0.35, *p* = 0.55). The means and standard error for each MDC are as follows: control–control, 2.25% ± 0.06%; flaxseed–control, 2.41% ± 0.08%; control–flaxseed, 2.50% ± 0.06%; and flaxseed–flaxseed, 2.58% ± 0.05%.

**FIGURE 3:**
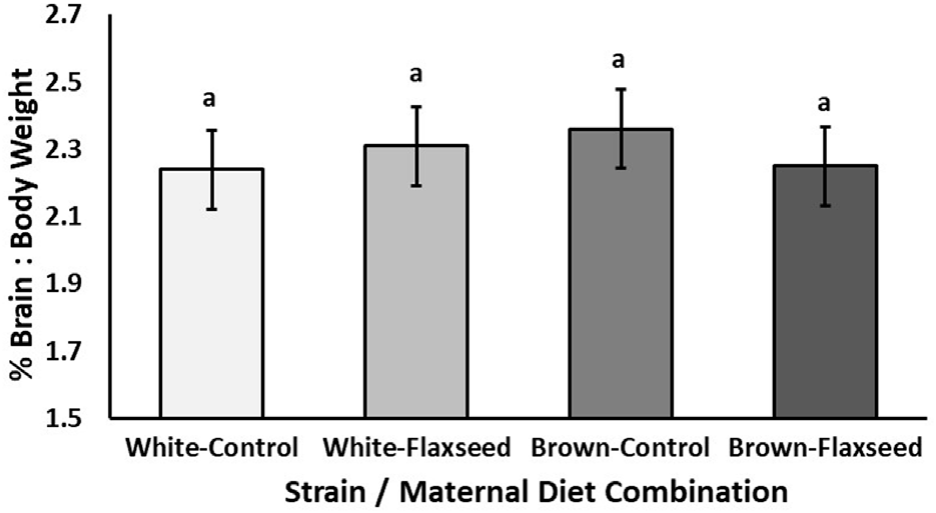
Experiment 2. Estimated mean (±SE) brain-to-body weight percentage [(brain/body weight) * 100] of day-old Shaver White and ISA Brown layer chicks from breeders fed either a control or flaxseed diet. The interaction between the strain and maternal diet tended to explain the brain-to-body weight percentage of the offspring (χ^2^ = 3.65, *p* = 0.056), but there were no significant pairwise comparisons (*p* > 0.28).

Experiment 2: For layer chicks, there tended to be an interaction between the strain and maternal diet (χ^2^ = 3.65, *p* = 0.056) for the brain-to-body weight percentage of layer offspring. However, there were no significant pairwise comparisons (Figure 3; *p* > 0.28). The means and standard error for each strain and diet combination are Brown–control, 2.36% ± 0.04%, Brown–flaxseed, 2.22% ± 0.05%, White–control, 2.24% ± 0.04%, and White–flaxseed, 2.31% ± 0.04%. There was no effect of strain (χ^2^ = 0.48, *p* = 0.49) or maternal diet (χ^2^ = 0.21, *p* = 0.65) alone on the brain-to-body weight percentage of layer chicks.

### 3.3 Brain fatty acids

Experiment 1: Analysis of brain fatty acids showed a significant effect of the laying diet on the µg ALA/g brain in broiler offspring brains. Broiler offspring from the control laying diet had a significantly higher concentration of ALA in the brain than offspring from the flaxseed laying diet (F = 5.86, *p* = 0.02). There was also an interaction between maternal rearing and laying diet of the percentage of ALA in the brain (F = 4.18, *p* = 0.05). *Post hoc* analyses showed that offspring from the flaxseed–flaxseed maternal diet combination had a significantly lower percentage of ALA in the brain than offspring from the flaxseed–control maternal diet (CIL = −0.19, CIH = −0.004, *p* = 0.04). The maternal diet had no other significant effects on brain fatty acid concentrations or percentage composition. Complete brain fatty acid analysis for broiler offspring is given in Table 2.

**TABLE 2:** Experiment 1. Brain fatty acid concentrations (µg/1 g) and composition (% of total fatty acids) from total lipid analysis. Brain tissue was sampled from the offspring of broiler breeder hens fed with and without flaxseed during the rearing or laying periods. Significant pairwise comparisons (*p* < 0.05) between maternal rearing and laying diets are indicated with different superscript letters.

Rearing diet Fatty acid↓/laying diet	Unit	Control Control	Flaxseed Control	Control Flaxseed	Flaxseed Flaxseed	Rearing	ANOVA Laying	Rearing*laying
Alpha-linolenic acid (C18:3 n-3)	µg/1 g %	8.70 ± 2.29 0.11 ± 0.02^**ab**^	8.44 ± 1.43 0.16 ± 0.02^**a**^	5.55 ± 1.35 0.11 ± 0.03^**ab**^	3.48 ± 1.12 0.06 ± 0.02^**b**^	*F = 0.79, p = 0.38* *F = 0.11, p = 0.75*	***F = 5.86, p = 0.02*** ***F = 4.20, p = 0.05***	*F = 0.28, p = 0.60* ***F = 4.18, p = 0.05***
Stearidonic acid (C18:4 n-3)	µg/1 g %	4.88 ± 0.43 0.07 ± 0.01	6.25 ± 2.32 0.10 ± 0.03	5.78 ± 2.25 0.09 ± 0.03	9.00 ± 2.79 0.13 ± 0.07	*F = 1.00, p = 0.38* *F = 1.37, p = 0.26*	*F = 0.57, p = 0.46* *F = 0.89, p = 0.36*	*F = 0.14, p = 0.72* *F = 0.01, p = 0.92*
Eicosatrienoic acid (C20:3 n-3)	µg/1 g %	5.33 ± 0.77 0.07 ± 0.01	4.10 ± 0.32 0.08 ± 0.03	3.83 ± 0.40 0.07 ± 0.03	4.71 ± 1.17 0.07 ± 0.04	*F = 0.02, p = 0.89* *F = 0.03, p = 0.86*	*F = 0.17, p = 0.69* *F = 0.49, p = 0.49*	*F = 1.33, p = 0.71* *F = 0.06, p = 0.81*
Eicosapentaenoic acid (C20:5 n-3)	µg/1 g %	16.06 ± 6.70 0.24 ± 0.10	5.26 ± 0.50 0.11 ± 0.45	4.97 ± 1.32 0.10 ± 0.02	8.77 ± 3.21 0.12 ± 0.04	*F = 0.65, p = 0.43* *F = 0.72, p = 0.86*	*F = 0.67, p = 0.42* *F = 1.00, p = 0.33*	*F = 3.27, p = 0.08* *F = 1.85, p = 0.19*
Docosapentaenoic acid (C22:5 n-3)	µg/1 g %	133.74 ± 48.09 1.63 ± 0.45	74.23 ± 16.90 1.57 ± 0.44	72.54 ± 15.21 1.37 ± 0.35	67.16 ± 12.84 1.20 ± 0.29	*F = 1.37, p = 0.25* *F = 0.12, p = 0.74*	*F = 1.32, p = 0.26* *F = 0.58, p = 0.45*	*F = 0.92, p = 0.35* *F = 0.02, p = 0.89*
Docosahexaenoic acid (C22:6 n-3)	µg/1 g %	872.64 ± 96.85 11.51 ± 0.56	683.52 ± 64.81 12.67 ± 0.57	658.11 ± 57.18 11.69 ± 0.71	810.61 ± 144.21 12.24 ± 0.63	*F = 0.01, p = 0.94* *F = 1.63, p = 0.22*	*F = 0.08, p = 0.79* *F = 0.05, p = 0.83*	*F = 2.12, p = 0.16* *F = 0.22, p = 0.64*
Σ n-3	µg/1 g %	1041.35 ± 134.03 12.63 ± 0.57	781.80 ± 64.81 14.68 ± 2.26	750.79 ± 61.05 13.44 ± 0.97	903.73 ± 144.21 13.82 ± 0.63	*F = 0.12, p = 0.73* *F = 0.61, p = 0.45*	*F = 0.30, p = 0.59* *F = 0.41, p = 0.53*	*F = 2.55, p = 0.13* *F = 0.12, p = 0.70*
Linoleic acid (C18:2 n-6)	µg/1 g %	157.60 ± 26.83 1.98 ± 0.10	118.63 ± 18.15 2.13 ± 0.14	113.64 ± 12.89 1.98 ± 0.08	124.05 ± 15.95 1.94 ± 0.04	*F = 0.46, p = 0.51* *F = 0.21, p = 0.65*	*F = 0.73, p = 0.40* *F = 0.99, p = 0.33*	*F = 1.43, p = 0.24* *F = 0.89, p = 0.36*
Gamma-linolenic acid (C18:3 n-6)	µg/1 g %	0.91 ± 0.55 0.01 ± 0.007	0.88 ± 0.40 0.02 ± 0.007	0.68 ± 0.40 0.01 ± 0.007	1.75 ± 0.92 0.02 ± 0.01	*F = 0.64, p = 0.43* *F = 0.84, p = 0.37*	*F = 0.24, p = 0.63* *F = 0.17, p = 0.69*	*F = 0.56, p = 0.11* *F = 0.56, p = 0.46*
Eicosadienoic acid (20:2 n-6)	µg/1 g %	60.04 ± 8.74 0.77 ± 0.02	46.46 ± 6.81 0.83 ± 0.04	45.02 ± 5.26 0.78 ± 0.08	49.10 ± 5.91 0.77 ± 0.55	*F = 0.39, p = 0.54* *F = 0.64, p = 0.34*	*F = 0.58, p = 0.46* *F = 0.58, p = 0.46*	*F = 1.44, p = 0.24* *F = 1.47, p = 0.24*
Eicosatrienoic acid (20:3 n-6)	µg/1 g %	39.96 ± 3.87 0.53 ± 0.03	28.34 ± 2.25 0.53 ± 0.04	28.83 ± 3.64 0.50 ± 0.03	37.36 ± 7.97 0.55 ± 0.03	*F = 0.03, p = 0.87* *F = 0.59, p = 0.45*	*F = 0.01, p = 0.94* *F = 0.05, p = 0.83*	*F = 2.66, p = 0.12* *F = 0.42, p = 0.53*
Arachidonic acid (20:4 n-6)	µg/1 g %	656.29 ± 99.62 8.38 ± 0.29	485.33 ± 64.52 8.72 ± 0.22	496.53 ± 59.74 8.61 ± 0.18	551.80 ± 83.70 8.52 ± 0.24	*F = 0.37, p = 0.55* *F = 0.18, p = 0.68*	*F = 0.20, p = 0.66* *F = 0.01, p = 0.98*	*F = 1.66, p = 0.22* *F = 0.68, p = 0.42*
Docosadienoic acid (22:2 n-6)	µg/1 g %	65.22 ± 24.30 0.83 ± 0.23^**a**^	92.52 ± 58.41 1.57 ± 0.88^**a**^	91.19 ± 33.56 1.66 ± 0.60^**a**^	26.52 ± 7.93 0.41 ± 0.13^**a**^	*F = 0.41, p = 0.53* *F = 0.34, p = 0.56*	*F = 0.40, p = 0.53* *F = 0.17, p = 0.68*	*F = 1.58, p = 0.22* *F = 3.10, p = 0.09*
Adrenic acid (C22:4 n-6)	µg/1 g %	213.06 ± 37.58 2.68 ± 0.15	152.74 ± 27.47 2.65 ± 0.21	177.71 ± 23.27 3.13 ± 0.31	158.60 ± 13.00 2.16 ± 0.18	*F = 2.03, p = 0.17* *F = 1.41, p = 0.25*	*F = 0.23, p = 0.63* *F = 0.64, p = 0.43*	*F = 0.55, p = 0.47* *F = 1.07, p = 0.31*
Docosapentaenoic acid (C22:5 n-6)	µg/1 g %	206.72 ± 71.22 2.43 ± 0.73	91.90 ± 24.89 1.48 ± 1.03	127.22 ± 32.65 2.14 ± 0.38	105.13 ± 23.08 1.90 ± 0.52	*F = 2.34, p = 0.14* *F = 1.00, p = 0.34*	*F = 0.46, p = 0.51* *F = 0.02, p = 0.88*	*F = 1.09, p = 0.31* *F = 0.40, p = 0.53*
Σ n-6	µg/1 g %	1399.80 ± 241.79 17.61 ± 0.96	1016.81 ± 159.85 17.93 ± 1.03	1080.83 ± 129.75 18.82 ± 0.38	1054.12 ± 126.12 16.74 ± 0.72	*F = 1.25, p = 0.28* *F = 1.06, p = 0.32*	*F = 0.51, p = 0.48* *F = 0.01, p = 0.94*	*F = 0.96, p = 0.34* *F = 1.62, p = 0.22*
Σ n-6:Σ n-3	µg/1 g %	1.32 ± 0.13 1.32 ± 0.13	1.27 ± 0.14 1.27 ± 0.14	1.46 ± 0.14 1.46 ± 0.14	1.25 ± 0.10 1.25 ± 0.10	*F = 0.94, p = 0.34* *F = 0.94, p = 0.34*	*F = 0.14, p = 0.71* *F = 0.14, p = 0.71*	*F = 0.36, p = 0.56* *F = 0.36, p = 0.56*

Bold values represent significant values at *p* ≤ 0.05.

Experiment 2: In the layer offspring, there was a significant effect of the maternal diet on the percentage of DHA (F = 20.97, p < 0.001), Σ n-3 (F = 26.91, p < 0.001), and Σ n-6 (F = 79.20, p < 0.001), with offspring from flaxseed-fed mothers having a higher percentage of DHA and Σ n-3, a lower percentage of Σ n-6, and a lower n-6:n-3 ratio. Table 3 shows that the maternal flaxseed diet increased the concentration and percentage of n-3 FAs with a corresponding decrease in n-6 FAs in layer offspring brains.

**TABLE 3:** Brain fatty acid concentrations (µg/1 g) and composition (% of total fatty acids) from total lipid analysis. Brain tissue was sampled from the offspring of ISA Brown and Shaver White layer breeder hens fed with and without flaxseed. Significant pairwise comparisons (*p* < 0.05) between strain and maternal diet treatment are indicated with different superscript letters.

Strain Fatty acid↓/maternal diet	Unit	Brown Control	Brown Flaxseed	White Control	White Flaxseed	Strain	*ANOVA* Diet	Strain*diet
Alpha-linolenic acid (C18:3 n-3)	µg/1 g %	7.03 ± 1.08 0.16 ± 0.02	8.60 ± 1.42 0.16 ± 0.01	6.93 ± 0.75 0.15 ± 0.02	8.83 ± 1.79 0.17 ± 0.04	*F = 0.00, p = 0.96* *F = 0.00, p = 0.98*	*F = 1.44, p = 0.22* *F = 0.36, p = 0.56*	*F = 0.01, p = 0.91* *F = 0.11, p = 0.74*
Stearidonic acid (C18:4 n-3)	µg/1 g %	3.09 ± 0.42 0.07 ± 0.005	3.97 ± 0.61 0.07 ± 0.008	3.34 ± 0.77 0.07 ± 0.013	3.56 ± 0.49 0.08 ± 0.008	*F = 0.01, p = 0.91* *F = 0.05, p = 0.84*	*F = 0.74, p = 0.40* *F = 0.19, p = 0.67*	*F = 0.26, p = 0.61* *F = 0.08, p = 0.78*
Eicosatrienoic acid (C20:3 n-3)	µg/1 g %	2.30 ± 0.47 0.05 ± 0.01	4.89 ± 1.08 0.09 ± 0.01	3.43 ± 0.77 0.07 ± 0.02	4.49 ± 0.31 0.09 ± 0.01	*F = 0.24, p = 0.63* *F = 0.48, p = 0.50*	***F = 5.97, p = 0.02*** ***F = 10.25, p = 0.004***	*F = 1.04, p = 0.32* *F = 0.67, p = 0.42*
Eicosapentaenoic acid (C20:5 n-3)	µg/1 g %	4.36 ± 0.23 0.10 ± 0.01^**a**^	6.41 ± 0.52 0.13 ± 0.01^**b**^	5.00 ± 0.83 0.10 ± 0.02^**a**^	7.99 ± 0.43 0.16 ± 0.01^**c**^	*F = 3.42, p = 0.08* ***F = 5.52, p = 0.03***	***F = 17.75, p<0.001*** ***F = 47.37, p<0.001***	*F = 0.61, p = 0.44* ***F = 5.68, p = 0.03***
Docosapentaenoic acid (C22:5 n-3)	µg/1 g %	18.20 ± 1.09 0.40 ± 0.02	26.71 ± 1.44 0.54 ± 0.04	23.57 ± 3.44 0.46 ± 0.02	25.97 ± 1.21 0.52 ± 0.02	*F = 1.08, p = 0.31* *F = 0.51, p = 0.48*	***F = 6.00, p = 0.02*** ***F = 13.27, p = 0.002***	*F = 1.98, p = 0.18* *F = 1.89, p = 0.19*
Docosahexaenoic acid (C22:6 n-3)	µg/1 g %	601.29 ± 28.18 13.24 ± 0.22	722.48 ± 71.64 14.06 ± 0.28	689.35 ± 96.56 13.40 ± 0.29	749.49 ± 26.34 14.92 ± 0.08	*F = 0.69, p = 0.42* *F = 4.00, p = 0.06*	*F = 1.72, p = 0.21* ***F = 20.97, p<0.001***	*F = 0.20, p = 0.66* *F = 1.88, p = 0.19*
Σ n-3	µg/1 g %	636.25 ± 29.04 14.01 ± 0.21	773.06 ± 76.09 15.05 ± 0.31	731.62 ± 101.43 14.24 ± 0.29	800.33 ± 29.33 15.92 ± 0.09	*F = 0.71, p = 0.41* *F = 4.36, p = 0.05*	*F = 1.98, p = 0.18* ***F = 26.91, p<0.001***	*F = 0.22, p = 0.65* *F = 1.88, p = 0.23*
Linoleic acid (C18:2 n-6)	µg/1 g %	90.16 ± 5.40 1.98 ± 0.04	112.37 ± 17.20 2.13 ± 0.17	105.23 ± 13.01 2.08 ± 0.07	102.97 ± 4.45 2.05 ± 0.05	*F = 0.05, p = 0.82* *F = 0.02, p = 0.88*	*F = 0.64, p = 0.43* *F = 0.97, p = 0.34*	*F = 0.97, p = 0.34* *F = 1.99, p = 0.17*
Gamma-linolenic acid (C18:3 n-6)	µg/1 g %	0.61 ± 0.35 0.01 ± 0.007	1.70 ± 0.39 0.03 ± 0.006	0.83 ± 0.34 0.02 ± 0.008	1.24 ± 0.36 0.02 ± 0.007	*F = 0.10, p = 0.76* *F = 0.00, p = 0.99*	*F = 3.59, p = 0.07* *F = 2.23, p = 0.15*	*F = 0.74, p = 0.40* *F = 1.09, p = 0.31*
Eicosadienoic acid (20:2 n-6)	µg/1 g %	38.62 ± 2.40 0.85 ± 0.02	42.52 ± 5.77 0.81 ± 0.02	41.34 ± 5.92 0.80 ± 0.03	38.79 ± 1.17 0.77 ± 0.01	*F = 0.01, p = 0.92* *F = 3.14, p = 0.09*	*F = 0.02, p = 0.89* *F = 1.84, p = 0.19*	*F = 0.46, p = 0.51* *F = 0.00, p = 0.97*
Eicosatrienoic acid (20:3 n-6)	µg/1 g %	25.90 ± 1.46 0.57 ± 0.01	33.99 ± 3.82 0.66 ± 0.03	22.29 ± 2.52 0.44 ± 0.01	26.06 ± 0.67 0.52 ± 0.01	***F = 4.72, p = 0.04*** ***F = 49.16, p<0.001***	***F = 4.98, p = 0.04*** ***F = 19.99, p<0.001***	*F = 0.66, p = 0.43* *F = 0.09, p = 0.77*
Arachidonic acid (20:4 n-6)	µg/1 g %	412.64 ± 24.95 9.04 ± 0.11	443.60 ± 52.79 8.54 ± 0.07	481.52 ± 59.98 9.46 ± 0.07	451.61 ± 14.06 9.00 ± 0.05	*F = 0.68, p = 0.42* ***F = 26.44, p<0.001***	*F = 0.00, p = 0.99* ***F = 32.30, p<0.001***	*F = 0.43, p = 0.52* *F = 0.06, p = 0.82*
Docosadienoic acid (22:2 n-6)	µg/1 g %	8.07 ± 0.22 0.18 ± 0.01	8.98 ± 1.51 0.17 ± 0.01	6.22 ± 0.92 0.13 ± 0.02	5.63 ± 0.61 0.11 ± 0.01	***F = 6.35, p = 0.02*** ***F = 10.93, p = 0.004***	*F = 0.02, p = 0.88* *F = 0.54, p = 0.47*	*F = 0.53, p = 0.48* *F = 0.01, p = 0.93*
Adrenic acid (C22:4 n-6)	µg/1 g %	115.85 ± 6.16 2.54 ± 0.03	120.68 ± 15.00 2.32 ± 0.03	123.86 ± 16.44 2.42 ± 0.03	110.69 ± 3.55 2.21 ± 0.03	*F = 0.01, p = 0.94* ***F = 15.12, p<0.001***	*F = 0.11, p = 0.75* ***F = 52.40, p<0.001***	*F = 0.50, p = 0.49* *F = 0.04, p = 0.84*
Docosapentaenoic acid (C22:5 n-6)	µg/1 g %	80.23 ± 7.42 1.75 ± 0.11	42.69 ± 5.42 0.82 ± 0.02	70.00 ± 7.79 1.40 ± 0.06	31.06 ± 1.85 0.62 ± 0.04	*F = 2.68, p = 0.12* ***F = 15.10, p<0.001***	***F = 32.80, p<0.001*** ***F = 144.0, p<0.001***	*F = 0.01, p = 0.92* *F = 1.19, p = 0.29*
Σ n-6	µg/1 g %	772.09 ± 46.33 16.92 ± 0.23	806.53 ± 101.19 14.48 ± 0.12	851.33 ± 104.83 16.74 ± 1.18	768.05 ± 23.21 15.30 ± 0.08	*F = 0.06, p=0.81* *F = 1.24, p = 0.28*	*F = 0.08, p = 0.78* ***F = 79.20, p<0.001***	*F = 0.48, p = 0.50* *F = 0.00, p = 1.00*
Σ n-6:Σ n-3	µg/1 g %	1.21 ± 0.03 1.21 ± 0.03	1.03 ± 0.02 1.03 ± 0.02	1.18 ± 0.03 1.18 ± 0.03	0.96 ± 0.01 0.96 ± 0.01	*F = 3.62, p = 0.07* *F = 3.62, p = 0.07*	***F = 55.12, p<0.001*** ***F = 55.12, p<0.001***	*F = 0.53, p = 0.48* *F = 0.53, p = 0.48*

Bold values represent significant values at *p* ≤ 0.05.

## 4 Discussion

We used two avian models, commercial broiler and commercial layer chickens, to determine whether maternal n-3 FAs transfer to the egg and accumulate differentially in the brain tissue of offspring from mothers fed flaxseed-supplemented diets *versus* mothers fed the control diet. We hypothesized that maternal-fed n-3 FAs would alter offspring brain FA composition and brain size. Our hypotheses were partially supported in that supplementing the maternal diet with flaxseed significantly increased brain size in broiler offspring but not layer offspring. However, we found that a maternal flaxseed diet significantly increased the percentage of DHA and reduced the n-6-to-n-3 ratio in the brains of layer chicks but not broilers. The current study highlighted distinct differences in development and nutrient utilization between meat and egg-producing chickens, suggesting differences in how maternal-fed flaxseed diets influence the offspring brain.

Layer and broiler breeders have divergent phenotypes. Compared to layer strains selected for high egg production, broiler strains have been selected for fast growth and muscle development. The management of breeder flocks varies to accommodate these selection criteria. One key difference in the management is that layer breeders are fed *ad libitum*. In contrast, broiler breeders are fed a quantitatively restricted diet to maintain growth targets and ensure that breeders reach sexual maturity. For this reason, it is logistically challenging to conduct an experiment on broiler and layer breeders simultaneously and in a manner that allows for direct comparisons.

The experimental design of these two experiments also differed. Broiler breeders were fed a flaxseed or control diet in the rearing and laying periods, so some breeders only experienced an n-3 FA diet during rearing or laying. The layer breeders, in comparison, were fed the n-3 FA or control diet throughout the experiment and experienced no switching of diets for the laying period. The decision to simplify the feeding regime in the layer breeder experiment was to account for using two strains of layer breeders. This difference in experimental design is another reason why the two experiments could not be directly compared.

There appeared to be value in feeding some broiler breeders flaxseed in only the rearing or laying period. The broiler breeder yolk FA analysis results suggest an accumulative effect of feeding n-3 FAs to breeders. The breeders fed flaxseed throughout rearing and laying deposited the highest percentage of DHA in the egg. We also showed that although broiler breeder diets were switched at 19 WoA, breeders fed n-3 FAs in the rearing period only still deposited a higher percentage of n-3 FAs in the egg at 30 WoA than those that were never fed flaxseed. This carry-over effect suggests that broiler breeder hens may deposit n-3 FAs from stores in their body for at least 10 weeks after switching to a control diet. This n-3 FA accumulation could add some cost-effective value to feeding flaxseed, where it may not be required to feed n-3 FAs consistently to maintain different n-3 FA profiles in the egg.

In broiler chicks, we found that maternal flaxseed rearing and maternal laying diet increased the brain-to-body weight ratio (larger brain size) in chicks. This finding supports our hypothesis that maternal-fed n-3 FAs would alter offspring brain size; however, the same result was not observed in layer offspring. These results contradicted those obtained by Ajuyah et al. (2003), who did not find differences in brain size due to feeding broiler breeders marine n-3 FAs. Research on neurogenesis in rats has shown negative impacts on brain size due to n-3 FA deficiency during development (Coti Bertrand et al., 2006). In this study, feeding broiler breeders diets supplemented with flaxseed, particularly in the laying diet, increased the brain-to-body weight ratio. This result is interesting because, across species, the brain-to-body weight ratio of birds correlates with cognition and intelligence (Lefebvre et al., 2004). In layer offspring, we found that a maternal flaxseed diet altered the brain n-6-to-n-3 FA ratio, increasing the concentration of n-3 FA and decreasing n-6 FA concentrations, thus supporting our hypothesis. The differences in brain size and n-6:n-3 FA ratio results between broiler and layer chick may be due to metabolic differences. Cherian (2015) summarized that early nutrition with n-3 FAs provides extra energy for developing embryos and could positively affect broiler health status and allow for resources to be allocated toward the development of the brain. Altering the brain characteristics of chicks could result in potential changes in behavior, gene expression, or neuroendocrine responses (Schmitz and Ecker, 2008; Calder, 2011; Vinot et al., 2011).

Corn and soybean commercial diets commonly fed to chickens are low in n-3 FAs. Diets of populations of feral chickens consist of a range of vegetation, seeds, insects, and human food waste, unlike the diets of commercially housed chickens (Ferrario et al., 2017). Populations of range-fed chickens in Greece, observed to eat fallen fruit, vegetation, and insects, produce eggs with an n-6-to-n-3 ratio of 1.30:1 (Simopoulos and Salem, 1989; De Meester, 2008). Additionally, the development of Columbus^®^ eggs by Belovo (Sint-Eloois-Vijve, Belgium) heralded the production of commercial “wild-type” eggs with an n-6-to-n-3 ratio of 1.03:1 (De Meester, 2008). These ratios close to unity (1:1) are similar to those found in the yolks of many species of wild precocial birds (Surai and Speake, 2008). They could reflect the ideal ratios of n-6 to n-3 FAs required for developing chicken embryos. It has even been suggested that an n-6 to n-3 FA ratio of 1:1 is ideal for human brain health (Simopoulos, 2011). Species of wild precocial birds have been shown to have higher n-6-to-n-3 FA ratios in the egg than captive counterparts fed grain-based diets (Surai and Speake, 2008). Grain-based diets result in egg n-6-to-n-3 FA ratios of 6.8:1 for pheasants, 27.2:1 for partridges, and 10.7:1 for ducks compared to their wild counterparts having ratios of 0.4:1, 3.6:1, and 1.9:1, respectively (Surai and Speake, 2008). Our results could translate to other precocial birds bred in captivity that are fed grain-based diets, and the implications for offspring brain development may not be limited to commercial chickens.

Extensive analyses of broiler and layer breeder egg yolks conducted by Akbari Moghaddam Kakhki and Kiarie (2021) and Thanabalan (2023), respectively, showed that diet formulations enriched with flaxseed fed to the broiler and layer breeders altered yolk fatty acid profiles. More specifically, the same flaxseed diet fed to the same strain of broiler breeders at an identical n-6-to-n-3 FA ratio as in our study (4.17:1) resulted in a yolk ratio of 4.86:1 in the egg, compared with the control diet (26:1) resulting in a ratio of 15.6:1 in the egg (Thanabalan, 2023). The layer breeder diets in our study may not have resulted in a large enough difference in the yolk fatty acid ratios to yield differences in brain size between the flaxseed fed (n-6-to-n-3 FA ratio of 5.31:1) and control fed (n-6-to-n-3 FA ratio of 14.71:1) breeders. Using diets identical to those fed in the study reported here, Akbari Moghaddam Kakhki and Kiarie (2021) reported that ISA Brown breeders fed flaxseed produced eggs with an n-6-to-n-3 FA ratio of 3.42:1 compared with those fed the control diet producing eggs with a ratio of 5.23:1. Similarly, the Shaver White breeders fed flaxseed in that experiment produced eggs with an n-6-to-n-3 FA ratio of 2.64:1 compared to those fed the control diet producing eggs with a ratio of 3.40:1 (Akbari Moghaddam Kakhki and Kiarie, 2021). However, the layer offspring from mothers fed flaxseed did have an altered brain n-6:n-3 FA ratio, which was not observed in the broilers. Strain differences in the amount of n-3 FA deposited in the egg and embryo uptake and utilization of n-3 FAs have been found, with Shaver White embryos performing better than ISA Brown embryos (Akbari Moghaddam Kakhki and Kiarie, 2021). In this study, we also found that the maternal flaxseed diet tended to have an effect on the brain n-6:n-3 FA ratio between ISA Brown and Shaver White offspring. Therefore, observing differences between layer and broiler chickens would be unsurprising.

The current study adds to the scarce fundamental knowledge about n-3 FAs and chicken brain development. To date, research on relative brain size has focused on the effects of domestication or the effect of selection for tameness (Agnvall et al., 2017; Gjøen et al., 2023) and tends to not assess the effect of maternal nutrition. Feeding flaxseed to breeders increases broiler offspring brain size and alters the FA composition of layer offspring. These results suggest that egg- and meat-type chickens may utilize maternal-fed nutrients differently. Future research should assess the effects of maternal n-3 FA supplementation on the behavior of their offspring, given that brain morphology and the composition of FAs can be influenced through maternal diets. These results might have important implications for chicken breeders fed diets typically low in n-3 FAs. However, broader implications may also exist for all captive bird species fed diets low in n-3 FAs that are not typical of the self-selecting diet of their wild counterparts.

## Data Availability

The datasets presented in this study can be found in online repositories. The names of the repository/repositories and accession number(s) can be found at: Borealis, the Canadian Dataverse Repository https://doi.org/10.5683/SP3/WSZBAS.
